# Impairment of endocytosis-related factors FNBP1L, ARHGAP24, and ATP6V1B1 increases HIV-1 entry into dendritic cells

**DOI:** 10.1128/jvi.02066-24

**Published:** 2025-03-03

**Authors:** Marija Janevska, Wojciech Witkowski, Jolien Vermeire, Marek Borowicz, Evelien Naessens, Hanne Vanderstraeten, Hans Nauwynck, Herman Favoreel, Bruno Verhasselt

**Affiliations:** 1 Department of Diagnostic Sciences, Faculty of Medicine and Life Sciences, Ghent University 26656 , Ghent, Belgium; 2 Department of Internal Medicine and Pediatrics, Faculty of Medicine and Life Sciences, Ghent University 26656 , Ghent, Belgium; 3 Department of Laboratory Medicine, Ghent University Hospital 26656 , Ghent, Belgium; 4 Department of Parasitology, Virology and Immunology, Faculty of Veterinary Medicine, Ghent University 26656 , Ghent, Belgium; University Hospital Tübingen, Tübingen, Germany

**Keywords:** HIV-1, viral entry, dendritic cells, endocytosis, FNBP1L, ARHGAP24, ATP6V1B1, cytoskeleton

## Abstract

HIV-1 infects several types of CD4+ cells. Among these, dendritic cells (DCs) are considered one of the first to encounter the virus upon sexual transmission. Expression of several restriction factors, of which SAMHD1 is well known, limits productive infection. Still, DCs are essential players in shaping adaptive immune responses that contribute heavily to the pathogenesis of HIV. Here, we set out to identify other factors that potentially contribute to the resistance of dendritic cells to HIV infection. Since endocytosis and the cytoskeleton impact HIV infection, we have put special emphasis on proteins implied in these pathways. In a selective, shRNA-mediated knockdown screen in primary monocyte-derived dendritic cells (MDDCs) infected with HIV in the presence of SAMHD1-disactivating Vpx containing virus-like particles, three proteins hampering HIV-1 infection were identified: FNBP1L, ARHGAP24, and ATP6V1B1. Findings of our research indicate that upon blocking of factors involved in endocytosis, increased viral entry is observed providing supportive evidence for endocytosis mostly being a dead-end entry pathway for HIV infection of MDDCs. Additional experiments show that changes in the cytoskeleton and endosomal pH that lead to impaired fluid-phase endocytosis and phagocytosis are responsible for these shifts in the phenotype observed.

**IMPORTANCE:**

Understanding how HIV-1 interacts with dendritic cells (DCs) is pivotal in deciphering early viral transmission and immune evasion but is subject to a long-standing controversy in HIV virology. Therefore, the identification of endocytosis-related host factors as barriers to productive infection in DCs emphasizes the role of endocytosis as a restrictive pathway for viral entry. By disrupting these processes, we highlight a shift in the cellular environment that could influence viral entry and transmission. These findings challenge existing models of HIV-1 entry into DCs. New insights into how cellular pathways limit viral spread have implications for the development of strategies aimed to curb viral dissemination and reservoir formation. Whether the knockdown of the proteins described simply augments the efficiency of infection via existing pathways or opens additional routes for HIV-1 entry remains to be investigated.

## INTRODUCTION

HIV-1 infects CD4+ cells, including T cells, macrophages, and dendritic cells (DCs). Positioned at mucosal surfaces, DCs are often among the first to encounter the virus during sexual transmission (1). As professional antigen-presenting cells (APCs), they patrol mucosal tissues sampling antigens and initiating immune responses, making them crucial in the early stages of HIV-1 infection (2–5).

While DCs are essential in shaping adaptive immune responses, their interaction with HIV-1 presents a paradox. Several outcomes of DC-HIV contacts are possible (6–9). DCs are not highly permissive to productive HIV-1 infection, a resistance attributed to various restriction factors, including SAMHD1 (10, 11), which blocks viral replication early in the infection process. Despite this, HIV-1 is capable of exploiting DCs to facilitate viral dissemination, as DCs can capture virions without productive infection, migrate to lymphoid tissues, and transfer virions to CD4+ T cells (8, 9, 12–16). This dual role—blocking productive infection while contributing to viral spread—underscores the complexity of HIV-1 interactions with DCs.

Since the primary function of DCs—capturing pathogens to present their antigens—is associated with endocytosis in a cytoskeleton-dependent manner, we decided to focus on host factors involved in these processes. However, the role of endocytosis in HIV-1 infection of DCs remains controversial. HIV-1 is known to hijack endocytosis and cytoskeleton pathways for trafficking and successful infection (17, 18). It is a question of debate, however, to what extent endocytosis drives initial virus-cell interactions. Depending on the cell model used, the importance of endocytosis can be crucial (19) or completely redundant (20). The regulation of endocytosis in DCs is closely linked to the actin cytoskeleton, which is responsible for driving membrane dynamics and vesicle formation and possibly viral entry (21, 22).

Here, we set out to investigate the contribution of cytoskeletal and endocytosis-related proteins to HIV-1 infection in primary, monocyte-derived dendritic cells (MDDCs) which are the most relevant *in vitro* model of immature DCs (23). Identifying these factors could provide deeper insights into the mechanisms that regulate endocytosis and viral trafficking in DCs, which is crucial for delineating the pathways that restrict HIV-1 infection. By identifying host proteins that limit viral entry, our study provides new insights into potential targets for antiviral strategies to limit HIV-1 infection, particularly during the earliest stages.

## RESULTS

### shRNA knock-down identifies factors involved in HIV-1 infection of dendritic cells

**Fig 1:**
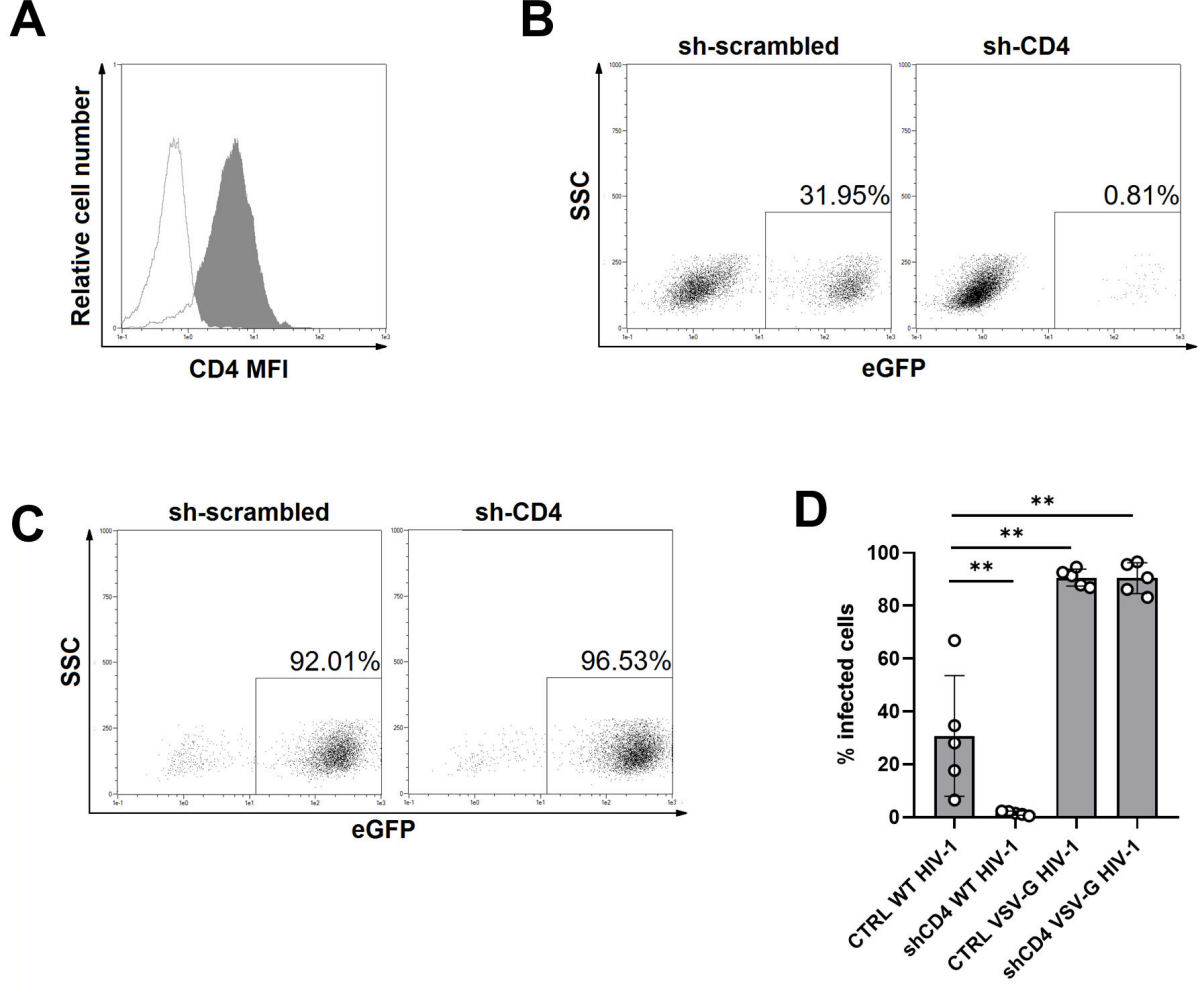
CD4 knockdown validates the screening approach. (A) Surface CD4 expression by flow cytometry on MDDC, day 5 post-transduction with vectors expressing control—non-targeting, scrambled shRNA (filled histogram), or CD4-targeting shRNA (empty histogram). Histogram depicts staining fluorescence intensity versus relative cell number. (**B)** Flow cytometry dot plots show MDDCs transduced with the shRNAs as indicated and infected with GFP-expressing CCR5-tropic HIV-1, on day 3 post-infection as measured by eGFP expression within the live population; figures represent the percentage of GFP+ cells (rectangle gate). (**C)** Flow cytometry dot plots show MDDCs transduced with the shRNAs as indicated and infected with VSV-G envelope pseudotyped HIV-1 and analyzed as in panel B. (**D)** Graph represents the percentage of infected cells on day 3 post-infection with CCR5 tropic HIV-1 or VSV-G pseudotyped env deleted counterpart, on either sh-scrambled (CTRL) or sh-CD4 transduced cells, as indicated. Individual data points shown, vertical lines indicate median, and standard deviation among the donors tested (*n* = 5, **P = 0.008).

In order to achieve effective knockdown of protein expression in monocyte-derived dendritic cells (MDDCs), we utilized a lentiviral shRNA transduction method developed in our lab (24). This technique, when used in combination with SAMHD1-degrading Vpx-containing Virus-Like Particles (VLPs) (25), allowed sufficient HIV-1 infection levels for reliable downstream analysis. As shown in Fig. 1A, shRNA targeting the CD4 receptor significantly reduced CD4 surface expression, as shown by flow cytometry. This knockdown inhibited HIV-1 infection, as illustrated by the decrease in eGFP-positive cells upon infection with a CCR5-tropic HIV-1 virus encoding eGFP (NL4-3-Bal-IRES-eGFP) (Fig. 1B). Conversely, the effect was bypassed when the virus was pseudotyped with the VSV-G envelope glycoprotein, indicating the specificity of the CD4-dependent entry pathway (Fig. 1C). Quantification of these effects (Fig. 1D) demonstrates a significant reduction in wild-type HIV-1 infection in cells expressing CD4-targeting shRNA, not observed when these cells were infected with VSV-G pseudotyped HIV-1 virus.

**Fig 2:**
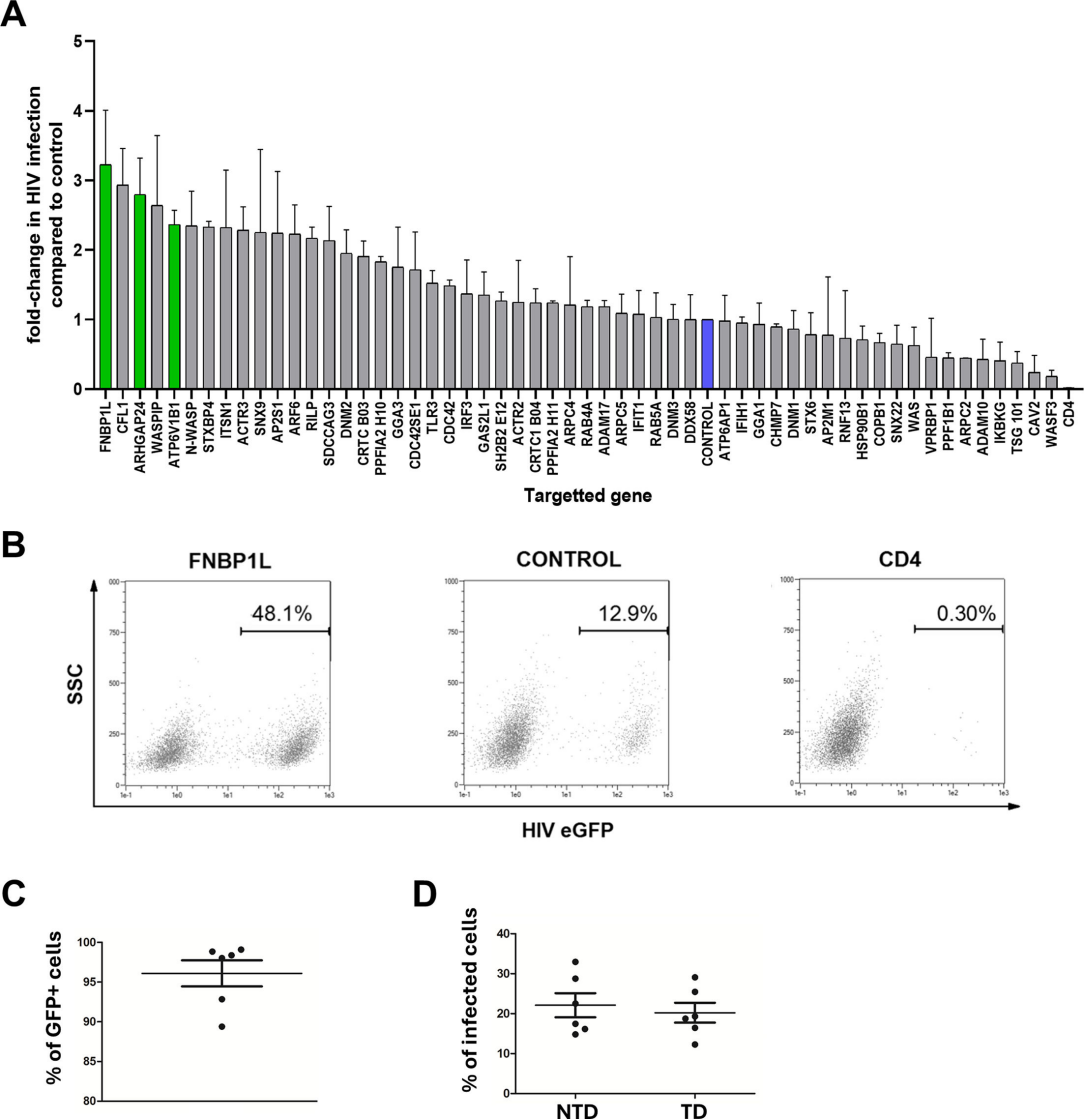
shRNA knockdown identifies factors involved in HIV infection of monocyte-derived dendritic cells. (A) Bar graph represents fold-change in HIV-1 NL4-3-Bal-IRES-eGFP infection rates (percentage eGFP+ cells) on day 3 post-infection in MDDCs transduced to express shRNA targeting the genes indicated in the presence of Vpx-containing VLPs. Results were normalized to the non-targeting, control scrambled shRNA transduced cells (depicted in blue). Error bars represent the standard deviation between the two donors tested. Green bars represent the factors selected for further validation. (**B)** Flow cytmetry dot plots show representative HIV-1 infection rates in cells from one of the donors used in the screening transduced to express scrambled (CONTROL) or targeting shRNA constructs (FNBP1L or CD4) as indicated. (**C and D)** Graphs show transduction rates of MDDCs from each of the donors used in the screening as verified by parallel transduction with the use of additional control vector encoding GFP (panel **C**) and comparison of HIV infection rates between non-transduced cells (NTD) and control transduced (TD) cells (with control vector used throughout the screening encoding non-targeting shRNA vector without fluorescent marker gene) (panel **D**).

Next, based on a literature search, we shortlisted cytoskeletal and endocytosis factors potentially involved in HIV-1 infection of MDDCs and assembled a panel of commercially available pLKO.1 shRNA lentiviral vectors from the RNAi Consortium (TRC 1 and 1.5). For each targeted gene, a single shRNA sequence was selected (as described in Table S1) based on validation data provided by the manufacturer. Each shRNA construct was used in two independent donors, and a total of six donors were used during the screening (Fig. 2AandB). For every donor, the cells were transduced separately in parallel with an eGFP-expressing control vector to verify transduction efficiency which was always high with an average exceeding 95% (Fig. 2C). Five days post-transduction, the MDDCs were infected with CCR5-tropic HIV-1 virus (NL4-3-Bal-IRES-eGFP). No differences in HIV-1 infection levels between the non-transduced and control scrambled shRNA-transduced cells were observed (Fig. 2D), illustrating that transduction did not affect HIV-1 infection.

As anticipated, CD4-targeting shRNA completely blocked HIV-1 infection, validating the screening approach. Knockdown of additional host factors, particularly caveolin-2 (CAV2), led to a notable reduction in infection by approximately fourfold, suggesting the involvement of caveolae as entry mechanism for HIV-1 into MDDCs that has already been previously reported (26–28). A number of other host factors seemed to hamper infection since their knockdown enhanced HIV-1 infection. Due to their behavior as restriction factors of HIV-1 infection in dendritic cells, we were especially interested in these factors. From this group, the actin cytoskeleton regulators FNBP1L and ARHGAP24, along with the vacuolar proton pump ATP6V1B1, were prioritized for further study due to their strong impact and lack of prior association with HIV-1 infection in the literature.

### FNBP1L, ARHGAP24, and ATP6V1B limit HIV-1 infection in MDDCs

**Fig 3:**
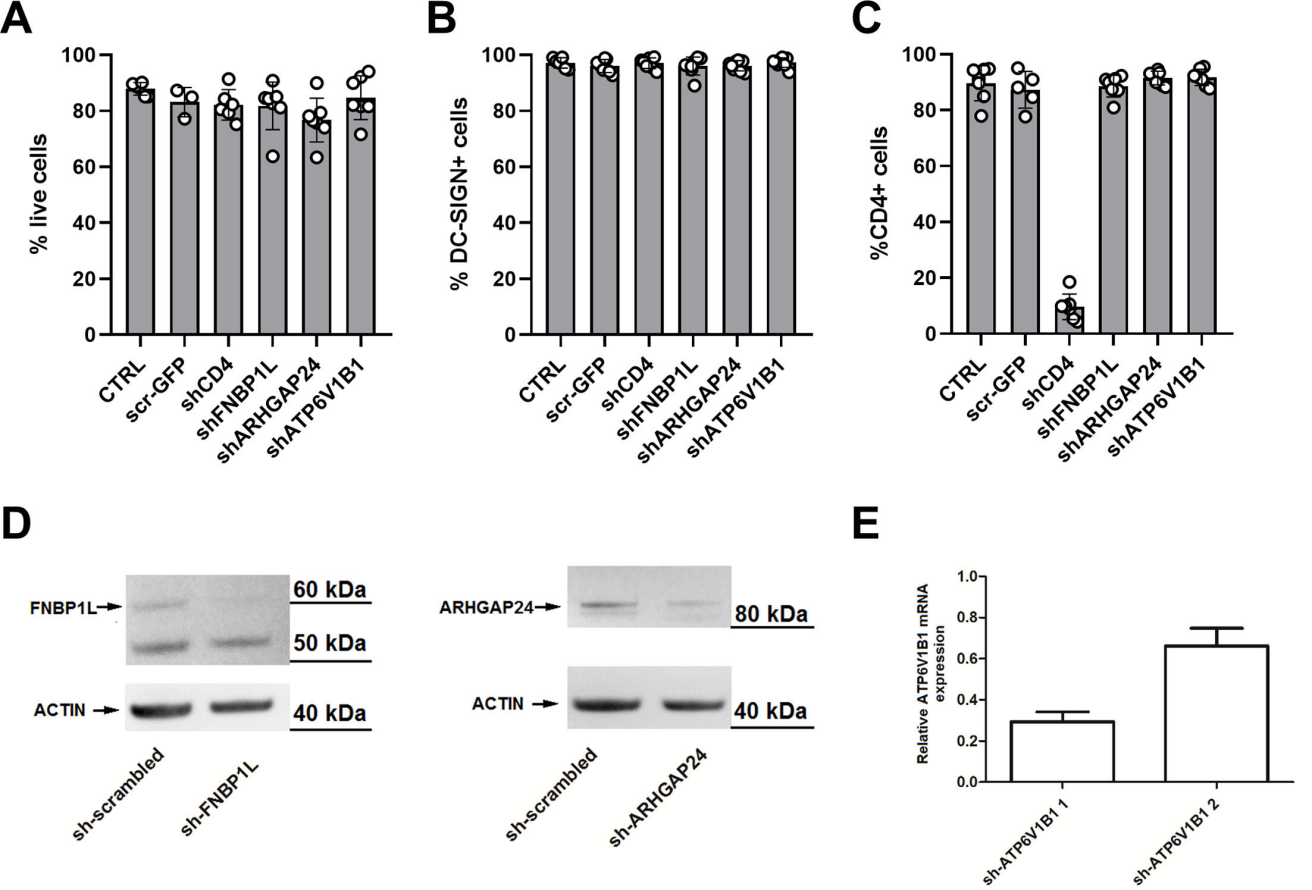
Experimental controls during HIV-1 infection of MDDCs cells expressing the indicated shRNA. (A) Bar graph depicting cell viability of MDDCs 5 days post-transduction with shRNA targeting CD4, FNBP1L, ARHGAP24, or ATP6V1B1. The percentage of live cells is presented, showing that transduction does not significantly affect cell viability (*n* > 5). (**B)** Bar graph showing expression of the differentiation marker DC-SIGN (CD209) in MDDCs 5 days post-transduction. Percentages represent DC-SIGN-positive cells (*n* > 5). (**C)** Bar graph displaying the percentage of CD4+ cells among MDDCs 5 days post-transduction with the indicated shRNA constructs (*n* > 5). (**D and E)** For each of the targeted factors, knockdown efficiency was quantified on protein (FNBP1L, ARHGAP24) or mRNA level (ATP6V1B1). Panel **D** shows a western blot analysis of FNBP1L and ARHGAP24 protein levels in MDDCs transduced with non-targeting control (sh-scrambled) or shRNA targeting each gene. Actin is shown as a loading control. Panel **E** shows a bar graph depicting the knockdown efficiency of ATP6V1B1 at the mRNA level, as quantified by RT-qPCR. Two shRNA vector were tested; however, only the vector depicted as sh-ATP6V1B1 1 was used for experiments. Data are presented as relative expression compared to control cells.

To confirm the screening results that identified these factors as potential modulators of HIV-1 infection, MDDCs from additional donors were transduced with shRNA targeting FNBP1L, ARHGAP24, or ATP6V1B1. Efficient transduction rates approaching 98%–99% were achieved for each donor, ensuring that the effects on HIV-1 infection could be accurately attributed to the gene knockdown. Figure 3A through C demonstrates that shRNA-mediated knockdown of these targets did not impair cell viability, differentiation (as indicated by DC-SIGN expression), nor CD4 protein expression. Protein levels of FNBP1L and ARHGAP24 were efficiently reduced, as shown by Western blot (Fig. 3D), while ATP6V1B1 knockdown efficiency was validated at the mRNA level due to the lack of validated antibodies (Fig. 3E).

**Fig 4:**
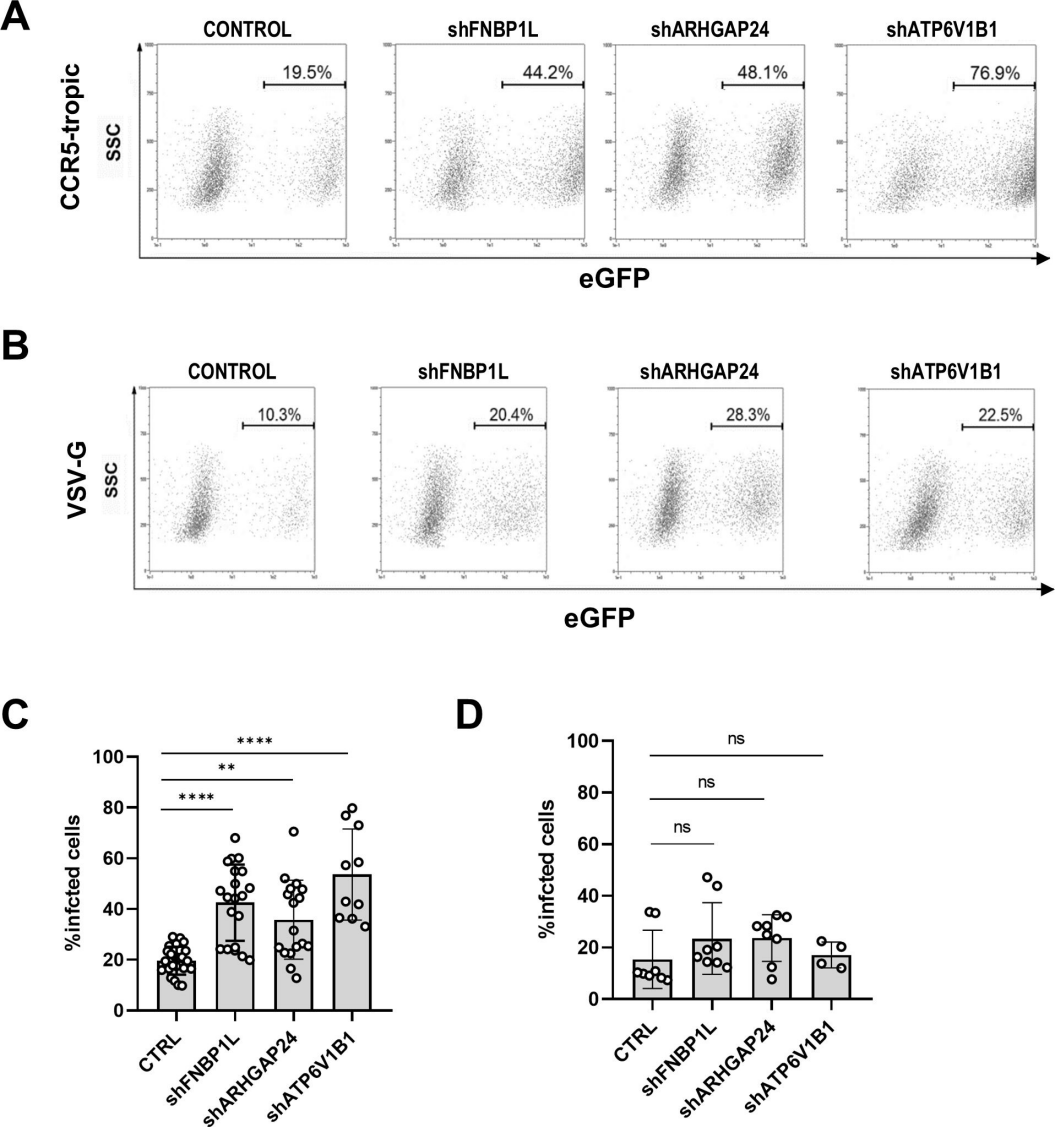
FNBP1L, ARHGAP24, and ATP6V1B knockdown in MDDCs increases HIV-1 infection, but this effect is not sustained upon VSV-G pseudotyping of the virus. (A) Flow cytometry plots show the percentage of eGFP+ MDDCs transduced to express the FNBP1L, ARHAGP24, and ATP6V1B1 shRNAs in the presence of Vpx-containing VLPs plotted against the side scatter (SSC), on day 3 post-infection with 50 ng p24 of HIV-1 NL4-3-Bal-IRES-eGFP (CCR5-tropic virus), compared to control transduced cells (CTRL) from a representative donor. (**B)** Flow cytometry plots show percentage of eGFP+ MDDCs transduced to express the FNBP1L, ARHAGP24, and ATP6V1B1 shRNAs in the presence of Vpx-containing VLPs plotted against the side scatter (SSC), on day 3 post-infection with 5 ng p24 of HIV-1 NL4-3-IRES-eGFP lacking WT envelope and pseudotyped with VSV-G, compared to control transduced cells (CTRL) from a representative donor. (**C and D)** Plots show infection levels (eGFP+ transduced MDDCs) infected with CCR5-tropic HIV-1-GFP (panel C, *n* ≥ 4, shARHGAP24 **P = 0.0023, shFNBP1L and shATP6V1B1 ****P < 0.0001) or with VSV-G pseudotyped HIV-1-GFP (panel D, *n* ≥ 4, shFNBP1L ns *P* = 0.2338, shARHGAP24 ns *P* = 0.1553, shATP6V1B1 ns *P* > 0.9999). Vertical lines represent the median and standard deviation among the tested donors.

FNBP1L, ARHGAP24, and ATP6V1B1 knockdown repeatedly proved to increase HIV-1 infection in additional donors, confirming screening observations (Fig. 4). When infected with wild-type, CCR5-tropic HIV-1, depletion of these factors significantly increased the proportion of eGFP-positive cells (Fig. 4A and C), confirming their roles in restricting HIV-1 infection. The effect had a similar trend but was not significant over donors when the virus was pseudotyped with VSV-G envelope and thus entered cells independently of the HIV-1 envelope (Fig. 4B and D), suggesting that the influence of these host proteins on viral infection is envelope-dependent. Notably, a reduced viral inoculum (5 ng p24) was used for the infection with the VSV-G pseudotyped virus compared to the wild-type virus (50 ng p24), given the greater permissiveness of MDDCs to VSV-G-mediated entry (as shown in Fig. 1D).

**Fig 5:**
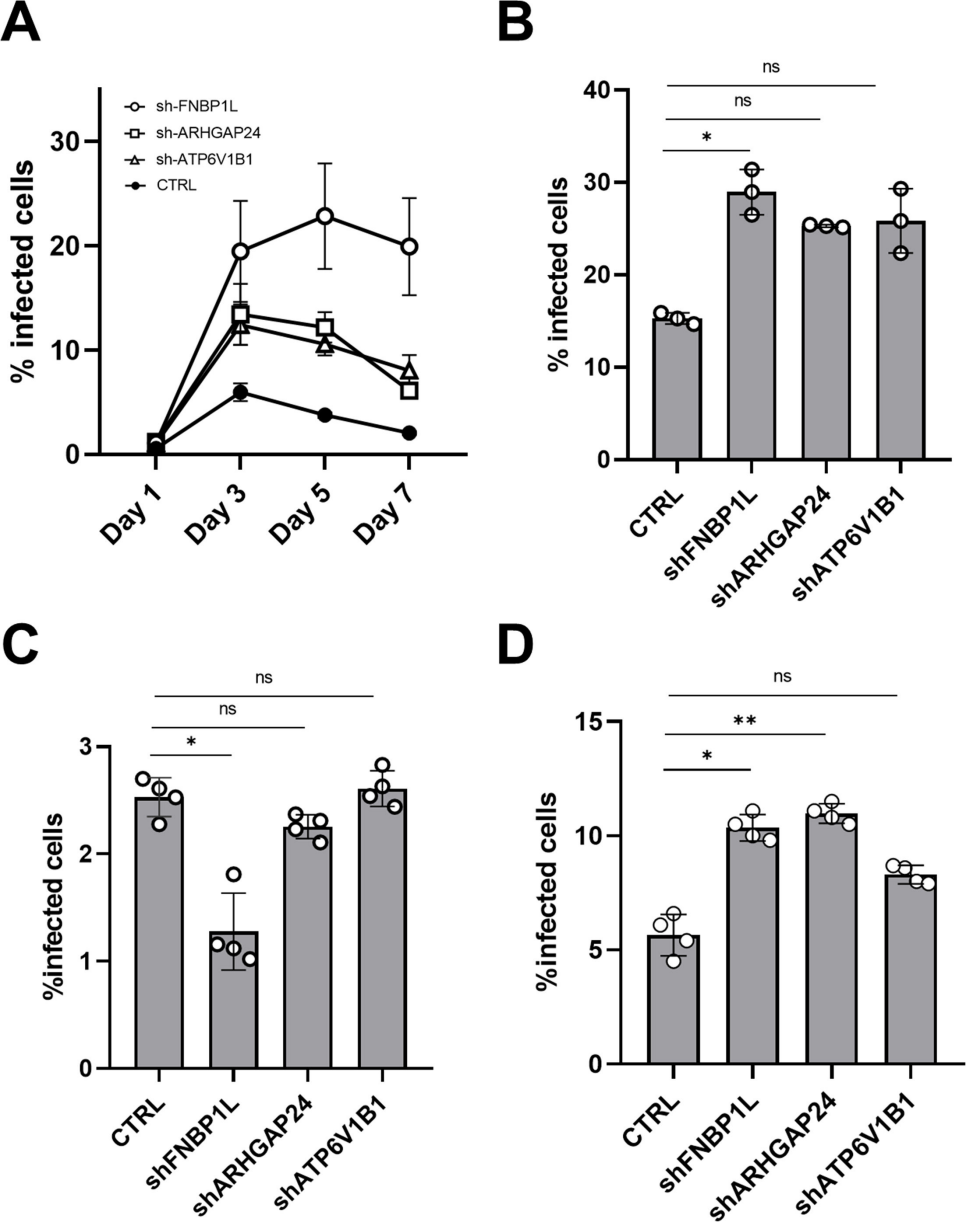
Effects of host protein knockdown on HIV-1 infection across cell types. (A) Line graph shows the effect of host protein knockdown on HIV-1 infection in THP1 cells over a 7-day period. Data represent viral infection levels at multiple time points post-infection compared to control cells (CTRL). (**B–D)** Bar graph depicts the effect of host protein knockdown on HIV-1 infection on day 3 post-infection in Jurkat CD4-CCR5 cells (panel **B**, *n* = 3, shFNBP1L *P = 0.0139, shARHGAP24 ns *P* = 0.5227, shATP6V1B1 ns *P* = 0.1627); in primary CD4+ T cells (panel **C**, *n* = 4, shFNBP1L *P = 0.0225, shARHGAP24 ns *P* = 0.0.5440, shATP6V1B1 ns *P* > 0.999); in monocyte-derived dendritic cells (MDDCs) treated with protease inhibitor (1 µM ritonavir) (panel **D**, *n* = 4, shFNBP1L *P = 0.0277, shARHGAP24 **P = 0.0.0025, shATP6V1B1 ns *P* = 0.7022). For panels B–D, Kruskal-Wallis ANOVA screening was performed to assess any preliminary differences between the groups.

To explore the broader relevance and specificity of these host proteins in HIV-1 infection, we extended our analysis to additional cell types. Further analysis confirms that these effects do not appear to be exclusive to MDDCs, as we also observe similar effects on HIV-1 infection when depleting these host factors in THP1 cells and Jurkat CD4-CCR5 cells, but not in primary CD4+ T cells (Fig. 5). Over a 7-day period in THP1 cells, knockdown of FNBP1L, ARHGAP24, and ATP6V1B1 resulted in sustained enhancement of HIV-1 infection (Fig. 5A). Similarly, in Jurkat cells, depletion of these factors increased HIV-1 infection rates (Fig. 5B). However, depletion of these host factors in primary CD4+ T cells seem to have no or even the opposite effect on HIV-1 infection suggesting a difference in the actin cytoskeleton dynamics of these cell types (Fig. 5C). Additionally, upon treatment of MDDCs with a protease inhibitor (ritonavir), and infection with HIV-1 in high MOIs (Fig. 5D), the same effect on HIV-1 infection was evident in single-rounds of infection. This and the fact that the influence of these host proteins on viral infection seems to be envelope-dependent, prompted us to investigate whether viral entry is one of the life cycle step that is restricted by these host factors.

### HIV-1 entry is restricted by FNBP1L, ARHGAP24, and ATP6V1B in MDDCs

**Fig 6:**
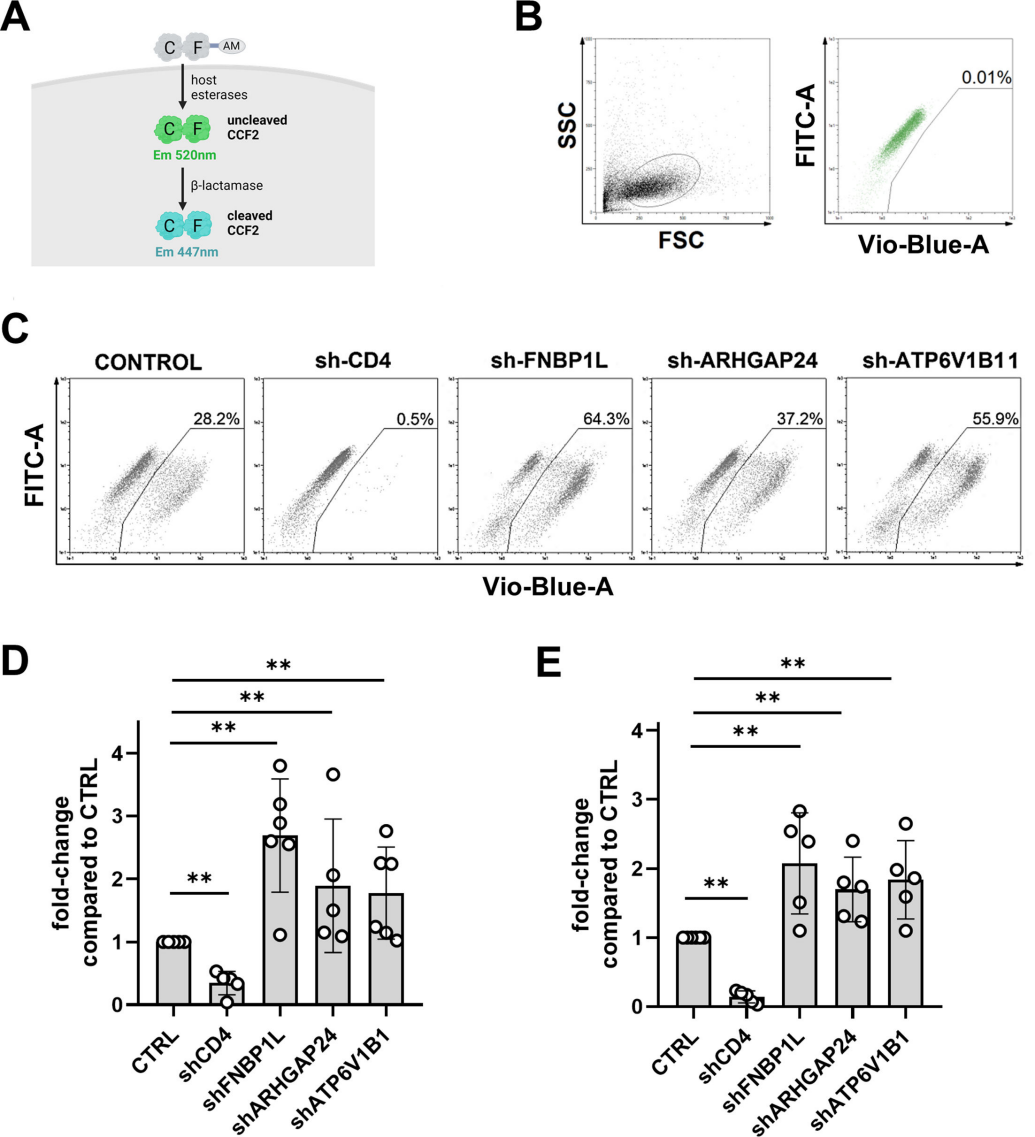
HIV-1 entry into transduced monocyte-derived dendritic cells. (A) Schematic representation of the BlaM-Vpr assay used to measure HIV-1 entry. Upon viral entry, host esterases cleave the AM tag, trapping the CCF2 substrate inside the cell. Once loaded, the green substrate gets cleaved by the β-lactamase (delivered by infecting the cells with HIV-1 virus that carries BlaM-Vpr fusion) causing a shift in fluorescence emission from green (520 nm) to blue (447 nm), indicating successful viral entry. (**B)** Gating strategy for identifying cleaved CCF2 in MDDCs. Dot plots show forward scatter (FSC) versus side scatter (SSC) and the fluorescence intensity of uncleaved (FITC channel) versus cleaved (VioBlue channel) CCF2 substrate in control, uninfected cells. MDDCs were loaded with CCF2 substrate on day 5 post-transduction and analyzed by flow cytometry after overnight incubation. (**C)** Representative flow cytometry plots show cleavage of CCF2 in MDDCs transduced with shRNAs targeting CD4, FNBP1L, ARHGAP24, or ATP6V1B1, following infection with BlaM-Vpr-containing HIV-1. The percentage of cells displaying viral entry is indicated, as demonstrated with a diagonal shift of the population upon cleavage of the CCF2 substrate (gated population). **(D and E)** Bar graphs show fold-change in viral entry compared to control (as shown in panel C) in MDDCs infected with CXCR4-tropic HIV-1 virus (panel **D**, *n* ≥ 4, shCD4 and shARHGAP24 **P = 0.0079, shFNBP1L and ATP6V1B1 **P = 0.0043) or with CCR5-tropic HIV-1 (panel **E,** *n* ≥ 4, shCD4, shFNBP1L, shARHGAP24, and shATP6V1B1 **P = 0.0022). Plots show individual data points, and error bars indicate standard deviation among the donors tested.

Given the nature of the proteins studied and their role in cytoskeletal organization and endocytosis, we postulated that these proteins could restrict HIV-1 entry in MDDCs. To investigate this possibility, we employed the BlaM-Vpr HIV entry assay (29–31) (Fig. 6A), a sensitive method for quantifying viral entry. In this assay, either CXCR4- or CCR5-tropic HIV-1 virions that contain β-lactamase protein (BlaM) fused to Vpr (BlaM-Vpr) are used for MDDCs infection. Fluorescence shift from green (520 nm) to blue (447 nm) marks viral entry and is visualized by flow cytometry as depicted in Fig. 6.

Depletion of FNBP1L, ARHGAP24, and ATP6V1B1 showed increased entry rates upon HIV-1 infection (Fig. 6C). As expected, knockdown of HIV-1 entry receptor CD4 dramatically decreased the ability of HIV-1 to enter the cells. Quantitative analysis of viral entry in MDDCs infected with CXCR4-tropic HIV-1 (Fig. 6D) and CCR5-tropic HIV-1 (Fig. 6E) shows that knockdown of FNBP1L, ARHGAP24, and ATP6V1B1 consistently leads to increased viral entry, irrespective of the virus coreceptor. The fold-change in entry was significant for both CXCR4 and CCR5-tropic HIV-1 viruses, reinforcing the hypothesis that the targeted host proteins play a restrictive role in the entry process of HIV-1 into MDDCs.

### Impaired endocytic activity in MDDCs following knockdown of host proteins suggests a shift in HIV-1 entry mechanism in MDDCs

To examine whether FNBP1L, ARHGAP24, and ATP6V1B1 knockdown affects endocytosis in MDDCs, we used two different functional endocytosis assays.

**Fig 7:**
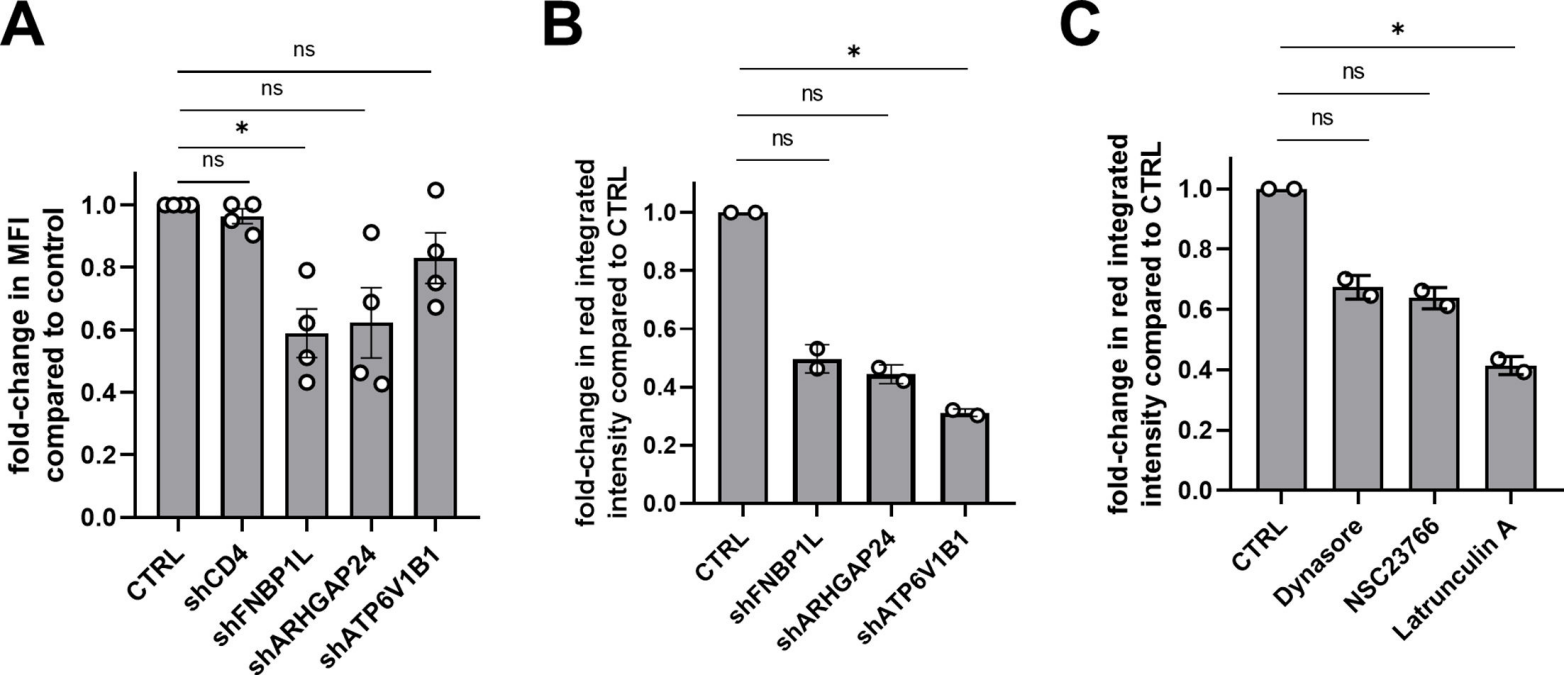
Assessment of endocytic activity in MDDCs following knockdown of host proteins. (A) Bar graph showing the effect of shRNA knockdown of CD4, FNBP1L, ARHGAP24, and ATP6V1B1 on FITC-dextran uptake by MDDCs. Data are presented as fold-change in mean fluorescence intensity (MFI) compared to control (CTRL), normalized to values obtained at 4°C to account for passive uptake (*n* = 4, shCD4 ns *P* = 0.9892, shFNBP1L *P = 0.0469, shARHGAP24 ns *P* = 0.0771, shATP6V1B1 ns *P* > 0.9999). (**B and C)** Graphs showing the effect of protein knockdown (panel **B**, shFNBP1L ns *P* = 0.9136, shARHGAP24 ns *P* = 0.4518, shATP6V1B1 *P = 0.0412) or treatment with endocytosis and cytoskeleton inhibitors as indicated (panel **C**, Dynasore ns *P* = 0.9136, NSC23766 ns *P* = 0.4518, Latrunculin A *P = 0.0412) on the phagocytosis of pHrodo *E. coli* bioparticles by MDDCs (*n* = 2). The fold-change in red integrated intensity, indicative of particle uptake and acidification, is calculated relative to control cells. For panels A-C, Kruskal-Wallis ANOVA screening was performed to assess any preliminary differences between the groups.

First, we assessed FITC-dextran uptake as a measure of fluid-phase endocytosis. As shown in Fig. 7A, knockdown of these proteins led to a reduction in endocytic activity, as indicated by a decrease in mean fluorescence intensity (MFI) compared to control cells. The effect was particularly notable with FNBP1L and ARHGAP24 knockdown, which resulted in the most pronounced reduction in FITC-dextran uptake, while depletion of the proton pump ATP6V1B1 only slightly impaired FITC-dextran uptake, as expected. CD4 knockdown, used as a control, shows no effect on the FITC-dextran uptake.

Second, we evaluated the effect of protein knockdown on the phagocytic uptake of pHrodo *Escherichia coli* bioparticles, which are designed to emit fluorescence upon acidification in endocytic compartments. Figure 7B shows that silencing FNBP1L, ARHGAP24, or ATP6V1B1 led to a decrease in integrated red fluorescene intensity, indicating reduced phagocytic activity. Interestingly, in contrast to the results of the FITC-dextran assay, depletion of the vacuolar proton pump ATP6V1B1 leads to significant reduction in pHrodo *E. coli* bioparticles uptake, confirming impaired acidification in the cells expressing shRNA targeting ATP6V1B1. This impairment in phagocytosis was replicated by treating the control cells with known endocytosis and cytoskeleton inhibitors (Dynasore, NSC23766, and Latrunculin A), which similarly decreased pHrodo bioparticle uptake and acidification (Fig. 7C), validating the assay. Quantified red integrated intensity values confirm these reductions, supporting the observed decrease in endocytic and phagocytic activities in MDDCs upon knockdown or inhibition of these factors.

## DISCUSSION

Our results show that the depletion of FNBP1L, ARHGAP24, and ATP6V1B1 enhances HIV-1 infection in MDDCs, but not in CD4+ T cells. Knocking down these key regulators of endocytosis reveals that a disrupted membrane environment and reduced vacuolar acidification allows the virus to enter MDDCs more efficiently.

FNBP1L (Toca-1) regulates actin by mediating membrane deformation, vesicle formation, and trafficking (32, 33). Its depletion impairs cell turgor, membrane ruffling and tubulation, processes essential for both macropinocytosis and receptor-mediated endocytosis (34, 35), both implicated in HIV-1 entry in DCs (36). Notably, FNBP1L also controls diaphanous-2 (DIAPH2)-dependent filopodia formation (37), which facilitates HIV-1 spread from infected dendritic cells (38). Possibly, FNBP1L mediates these effects through its interactions with Cdc42 and N-WASP-WIP complex. Interestingly, MDDCs transduced with shRNA targeting N-WASP (WASL), WIP (WASPIP) and CDC42 also exhibited increased susceptibility to HIV-1 infection (Fig. 2).

ARHGAP24 (FilGAP) is a GAP (GTPase-activating protein) for Rac1, a small Rho GTPase that acts as a “turn off” switch in the cell and regulates actin polymerization; the absence of ARHGAP24 leads to uncontrolled Rac1 hyperactivation which causes excessive membrane protrusions and impaired endocytosis (39–41). Persistent ruffles and lamellipodia interfere with vesicle closure, affecting clathrin-independent endocytosis and macropinocytosis (42, 43). MDDCs transduced with shRNA targeting both ARHGAP24 and ARF6 (another small GTPase) showed increased susceptibility to HIV-1 infection (Fig. 2). ARF6 is a coordinator that as part of the downstream signaling helps fine-tune Rac1 activity by prepping the membranes and correctly localizing Rac1 to the specific sites. Therefore, disruption in ARF6 can lead to uncontrolled Rac1 activation in aspecific sites, amplifying membrane ruffling, impairing endocytic processes and most likely leading to failure of endosome closure and maturation, and alternative viral entry (35, 44). While ARHGAP24 acts as a “brake” and ARF6 controls the correct localization of activated Rac1, depletion of both ARHGAP24 and ARF6 would affect Rac1 activity in a different manner.

ATP6V1B1, a subunit of V-ATPase, is critical for vacuolar acidification and endosome maturation (45, 46). Its depletion disrupts endosomal acidification, which impairs endosome maturation and lysosomal degradation. While endocytosis still occurs without ATP6V1B1 (as shown by FITC-dextran uptake, Fig. 7A), the lack of acidification could allow endocytosed HIV-1 to escape degradation, enhancing infection (47).

Langerhans cells, macrophages, and dendritic cells share a similar ability to capture HIV-1 and influence its transmission, but their roles differ based on unique receptor expression and processing mechanisms. A key distinction between DC subsets lies in the roles of their lectin receptors in HIV-1 processing. Langerin, expressed on Langerhans cells (LCs), captures HIV-1 and routes it to Birbeck granules for autophagic degradation, thereby restricting infection (48). This TRIM5α-dependent restriction is unique to LCs and is not observed in DCs, where DC-SIGN plays a different role. Unlike Langerin, DC-SIGN facilitates HIV-1 infection and transmission by abrogating TRIM5α restriction, allowing HIV-1 to evade degradation and be transferred to CD4+ T cells (49). This underscores how DCs act as facilitators of viral dissemination by bypassing certain restriction mechanisms.

The enhanced HIV-1 entry observed in the BlaM-Vpr assay (Fig. 6) following the knockdown of FNBP1L, ARHGAP24, and ATP6V1B1 suggests these factors restrict viral entry in MDDCs, by targeting endocytosed virus for subsequent lysosomal degradation. Our endocytosis assays show that depletion of these factors disrupts fluid-phase endocytosis and destructive phagocytosis, leading to reduced uptake of FITC-dextran and pHrodo *E. coli* bioparticles (Fig. 7A Through D). This disruption of the highly controlled membrane environment in dendritic cells likely facilitates an alternative, less restricted entry mechanism for HIV-1. Since DCs constantly sample the environment by endocytosis, it is likely that HIV virions present in their vicinity are taken up in that manner. Subsequently, the low pH of the endosome reduces HIV infectivity. Possibly, once endocytosis is impaired, more virions can enter upon fusion at the plasma membrane leading to productive infection as previously seen in CD4+T cells (50, 51).

In a recent study on HIV-1 entry in CD4+ T cells, however, this long-known entry pathway was challenged. It seems that HIV-1 preferentially fuses with pH-neutral endosomes rather than the plasma membrane or acidic endosomes in CD4+ T cells in a dynamin-2-dependent manner, as demonstrated by Sharma et al. (52). Though we did not test the effect of DNM2 knockdown on HIV-1 infection in CD4+ T cells, the increase in infection observed upon DNM2 knockdown in MDDCs in our study (Fig. 2A) suggests cell-specific differences in the reliance on dynamin-2-mediated endocytosis. Further studies are necessary to characterize how HIV-1 entry mechanisms vary across immune cell types.

Furthermore, knockdown of the targeted host factors did not enhance infection with VSV-G pseudotyped HIV-1, highlighting the distinct entry mechanisms of VSV-G and wild-type HIV-1 (Fig. 4B and D). VSV enters via clathrin-mediated endocytosis through the LDL receptor, a pathway largely independent of actin dynamics (53, 54). In contrast, HIV-1 relies on more actin-dependent pathways like macropinocytosis or caveolar endocytosis, underscoring the role of the tightly controlled actin cytoskeleton in regulating HIV-1 entry in DCs (55).

Knocking down FNBP1L, ARHGAP24, and ATP6V1B1 in CD4+ T cells did not enhance HIV-1 infection, contrasting sharply with the significant effects observed in MDDCs (Fig. 5C). In fact, disturbing the actin cytoskeleton in CD4+ T cells by depleting FNBP1L seems rather to impair the viral entry. The differential effects of these knockdowns in MDDCs versus CD4+ T cells underscore the distinct entry pathways favored by HIV-1 in different cell types. It has been previously demonstrated that membrane tension determines the fate of the HIV-1 virions, as high membrane tension tends to be restrictive to viral entry (56). Dendritic cells have a denser actin cytoskeleton and several surface molecules with higher affinity for the HIV-1 viral envelope than the CD4 receptor that capture HIV in surface-bound compartments, making endocytosis an important restriction mechanism in DCs (16, 57). Our results further show that the host factors FNBP1L, ARHGAP24, and ATP6V1B1 restrict viral entry in a co-receptor-independent manner, as both CCR5- and CXCR4-tropic viruses equally show increased viral entry upon knockdown of these host factors (Fig. 6).

In summary, our study reveals that FNBP1L, ARHGAP24, and ATP6V1B1 restrict HIV-1 entry in MDDCs. We hypothesize this restriction works in two ways: (1) by pH-dependent endocytic uptake, trafficking, and degradation of the virus and (2) by maintaining a high-tension environment at the cell surface, which tightly regulates cellular trafficking and import of foreign particles in these professional antigen-presenting cells. Their impairment reroutes HIV-1 entry toward a more efficient entry pathway, possibly fusion. Future mechanistic studies investigating how these factors regulate viral entry will further deepen our understanding of HIV-1 pathogenesis and the role of dendritic cells and how they differ from other immune cells, potentially revealing new targets for antiviral interventions.

## MATERIALS AND METHODS

### Plasmids

The shRNA pLKO.1-puro plasmids were purchased as bacterial glycerol stocks from Sigma Aldrich (St. Louis, MO). When no validated constructs were available, those with the highest calculated efficiency were selected. A list of all the clones used can be found in Table S1. Replication competent, NL4-3-Bal-IRES-eGFP HIV-1 virus containing Bal CCR5-tropic envelope was generated as described previously (24) from proviral plasmid NLENG1-IRES engineered to express eGFP and kindly provided by Dr. D.N. Levy, New York University College of Dentistry, New York, NY (58, 59). NL4-3-IRES-HSA and its Bal *env* encoding counterpart constructs were kindly donated by Dr. M.J. Tremblay (Faculté de Médecine, Université Laval, Québec, Canada) (60). For the VSV-G pseudotyped HIV, NL4.3 proviral construct—a kind gift from Dr. F. Kirchhoff, Institute of Molecular Virology, Ulm University Medical Center, Germany—encoding eGFP and containing a defective *env* gene (61) was used in viral production together with vesicular stomatitis virus envelope plasmid pMD.G (62). SgpΔ2 SIV-based vector for the production of Vpx-VLP is a kind gift from Dr. K. Überla (Department of Molecular and Medical Virology, Ruhr-University-Bochum, Germany) (63). The plasmid encoding β-lactamase linked to the N terminus of Vpr (BlaM-Vpr) and pAdVAntage vector were kindly provided by Dr. O.T. Fackler (Department of Virology, University of Heidelberg, Germany) (64).

### Monocyte isolation and lentiviral transduction

Monocytes were isolated from buffy coats of healthy donors following the previously described protocol (24). MDDCs are generated by culturing monocytes with 500 IU/mL IL4 and 1,000 IU/mL GM-CSF for 6 days. Lentiviral vector supernatants were produced from pLKO.1-puro vectors as reported (64). Lentiviral transduction was performed on day 1 post-monocyte isolation in the presence of Vpx-containing VLP, polybrene and with spinoculation according to the established method (24).

### Production of Vpx-VLP and HIV viruses

NL4-3-Bal-IRES-eGFP and Vpx-VLP were produced by transfecting 293T cells as described previously (24). VSV-G pseudotyped NL4.3 virus production followed a similar protocol. In order to produce Blam-Vpr containing HIV particles used for the viral entry assay, the NL4-3-IRES-HSA plasmid was co-transfected into the 293T cells together with BlaM-Vpr and pAdVAntage vectors according to manufacturer’s instructions (JetPei Polyplus, Sélestat, France). Plasmid ratios were calculated following the guidelines described by Cavrios et al (31). Supernatant was refreshed 24 h and collected 48 h post-transfection. Following 10 min centrifugation at 900 g to pellet cellular debris, the viral supernatant were concentrated using Amicon Ultra Filters. Subsequently, the concentrated supernatant was aliquoted and stored at −80°C. All HIV viral supernatants were titrated by measurement of viral reverse transcriptase (RT) activity and expressed as equivalent of p24 (65).

### HIV infection and entry assay of MDDCs

Six days post-transduction the cells were plated in a flat-bottom 96-well plate at 50,000 cells/well in the final 200 µL of RPMI complete growth medium (containing P/S antibiotics and 10% FCS) and infected (50 ng p24 for non VSV-G and 50 or 5 ng p24 for VSV-G pseudotyped HIV virions) by spinoculation (90 min, 950 g, 32°C). On day 1, post-infection medium was refreshed. Infection was measured on day 3 by flow cytometry, gating on eGFP expressing, live cells as judged by propidium iodide staining (Miltenyi Biotec, Leiden, Netherlands). HIV BlaM-Vpr infection of transduced MDDCs followed protocol identical to the one described above (50 ng p24/well). After spinoculation, cells were kept for 4 h at 37°C in a humidified atmosphere containing 5% (vol/vol) CO_2_. Next, the cells were washed once with warm RPMI complete growth medium, subsequently stained with the β-lactamase loading solution containing the CCF2 dye (Life Technologies), and incubated overnight at room temperature in the dark. Cells were then processed and analyzed as described (29–31, 66).

In a similar manner THP1 cells, Jurkat CD4-CCR5 cells and CD4+ T cells were infected and analyzed via flow cytometry 3 days post-infection.

### FITC-dextran and pHrodo *E. coli* bioparticles uptake

MDDCs were harvested and seeded at a concentration of 1 × 10^6 cells/mL in RPMI growth medium supplemented with 10% FBS. Cells were then incubated with 1 mg/mL FITC-dextran (70 kDa, Invitrogen, Waltham, MA, USA) at 37°C for 1 hour to allow for endocytosis. As a control, a parallel set of cells was incubated with FITC-dextran at 4°C to account for passive uptake. After incubation, cells were washed and then fixed for 15 minutes at room temperature. Fixed cells were analyzed using a flow cytometer (MACS Quant 10, Miltenyi Biotec), and the mean fluorescence intensity (MFI) was measured to assess dextran uptake. Data were normalized to the 4°C control to account for non-specific binding.

MDDCs were seeded in 96-well plates at a concentration of 1 × 10^5 cells per well in RPMI growth medium supplemented with 10% FBS. Cells were then treated with pHrodo Red *E. coli* Bioparticles (Thermo Fisher, Rockford, IL) as per the manufacturer’s instructions and incubated in the Incucyte live-cell imaging system (Sartorius, Göttingen, Germany) at 37°C for real-time monitoring of phagocytosis. Fluorescence images were captured every 15 minutes over a 2 hour period to monitor the red fluorescence emitted by the acidified phagocytic vesicles. The Incucyte software was used to calculate the average red integrated intensity (RCU × μm²/mm²), which reflects particle uptake and acidification. Quantitative data were normalized to control transduced cells.

### Flow cytometry and immunoblotting

For surface CD4 expression analyses, shRNA expressing MDDCs were stained with monoclonal mouse anti-human CD4-APC (M-T466, Miltenyi Biotec) and analyzed by flow cytometry (MACS Quant 10, Miltenyi Biotec). For immunoblotting, six days post-transduction, cells washed twice in PBS were lysed in Laemmli sample buffer and equal amounts of proteins were run on NuPAGE Novex 4%–12% Bis-Tris pre-cast polyacrylamide gels (Invitrogen) in reducing conditions. Proteins were blotted on polyvinylidene fluoride membranes (Invitrogen). Blots were probed with non-commercial, monoclonal mouse anti-FNBP1L (also known as Toca-1 and kindly provided by Dr. Giorgio Scita, IFOM, Milan, Italy) as previously described (67), polyclonal rabbit anti-human ARHGAP24 (ab84046, Abcam, Cambridge, UK) as previously shown (68), and monoclonal mouse anti-beta actin loading control antibody (BA3R, Thermo Scientific). Antibody complexes were detected by horseradish peroxidase conjugated anti-mouse or anti-rabbit IgGs (GE Healthcare, Diegem, Belgium) and revealed by enhanced chemiluminescence (Thermo Scientific). Protein expression levels were quantified relative to the beta actin loading control using the ImageJ software.

### Quantitative real-time PCR

Quantitative real-time PCR (qPCR) was used to measure the residual expression level of *ATP6V1B1* gene in MDDCs expressing the shRNA clones. Protocol previously described from our laboratory was applied (69) with YWHAZ (tyrosine 3-monooxygenase/tryptophan 5-monooxygenase activation protein) FWD primer (sense) 5′-ACTTTTGGTACATTGTGGCTTCAA-3′, REV primer (antisense) 5′-CCGCCAGGACAAACCAGTAT-3’, and RPL13A (Ribosomal Protein L13a) FWD primer (sense) 5′-CCTGGAGGAGAAGAGGAAAGAGA-3′, REV primer (antisense) 5′-TTGAGGACCTCTGTGTATTTGTCAA-3′ serving as reference genes, selected with the geNorm algorithm based on their expression stabilities (70). For *ATP6V1B1*, PrimePCR Sybr Green assay was used (qHsaCID0036438, Biorad, Nazareth, Belgium). Reference gene normalized *ATP6V1B1* expression relative to the non-targeting scrambled control was calculated with the qBase software (Biogazelle, Belgium).

### Statistical analysis

Nonparametric statistical analyses were conducted using GraphPad Prism version 8.00 for Windows (GraphPad Software, San Diego, CA). Specifically, the Mann-Whitney *U* test and Friedman test were used when appropriate (e.g., Fig. 1D, 4C, D, 6D and E), depending on the number of donors and the relevance for statistical significance to support the conclusions. To assess the overall difference between multiple groups and detect potential patterns, a Kruskal-Wallis test was performed as preliminary screening (e.g., Fig. 5B through D, 7B and C when *n* < 5).

## Data Availability

There are no large data sets. Many graphs show individual data points and raw data are available upon reasonable request.

## SUPPLEMENTAL MATERIAL

Supplemental material jvi.02066-24-s0001.docx Figure S1, experimental set-up overview; Table S1, shRNA sequences used.

ASM does not own the copyrights to Supplemental Material that may be linked to, or accessed through, an article. The authors have granted ASM a non-exclusive, world-wide license to publish the Supplemental Material files. Please contact the corresponding author directly for reuse.
