# Supplementary material for: Impairment of endocytosis-related factors FNBP1L, ARHGAP24, and ATP6V1B1 increases HIV-1 entry into dendritic cells
Source: J Virol. 2025 Mar 3;99(4):e02066-24. doi: 10.1128/jvi.02066-24 (PMC11998494; doi:10.1128/jvi.02066-24)
Supplement: Supplemental material — Figure S1, experimental set-up overview; Table S1, shRNA sequences used. [file jvi.02066-24-s0001.docx]

**
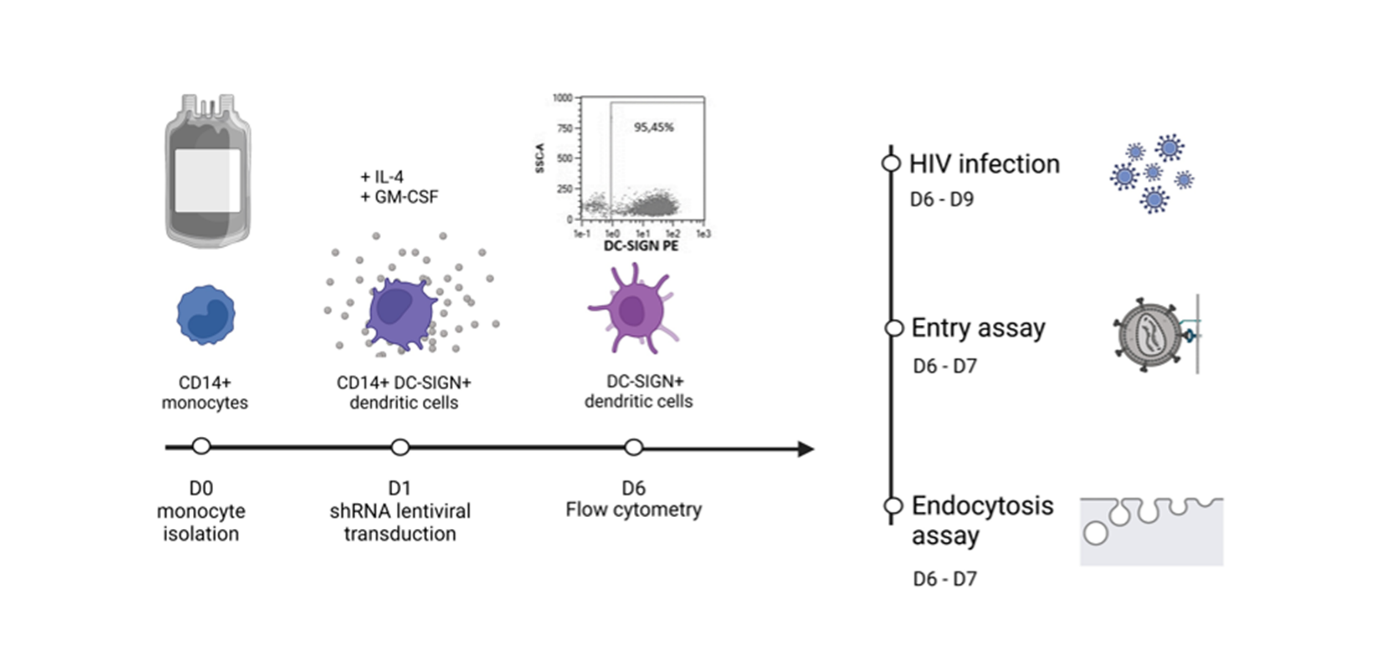
*Supplemental Figure 1*** ***Experimental set-up overview: MDDC differentiation and downstream viral and cellular assays.*** *CD14+ monocytes were isolated from peripheral blood and differentiated into DC-SIGN+ dendritic cells (DCs) through culture with IL-4 and GM-CSF. On day 1 (D1), cells were transduced with shRNA lentiviral vectors targeting specific host genes. By day 6 (D6), DCs were evaluated for DC-SIGN expression via flow cytometry, confirming successful differentiation. After confirming DC-SIGN positivity and cell viability, cells were used in various assays. HIV-1 infection assays were conducted between days 6 and 9 to assess the impact of gene knockdown on viral infection and replication. Blam-vpr fusion entry assay was performed on days 6 to 7 to evaluate the effect of knockdown on HIV-1 entry into MDDCs. Two different functional endocytosis assays (FITC-dextran assay and pHrodo E.coli assay), also performed on days 6 to 7, examined the involvement of targeted proteins in cellular uptake processes.*

**
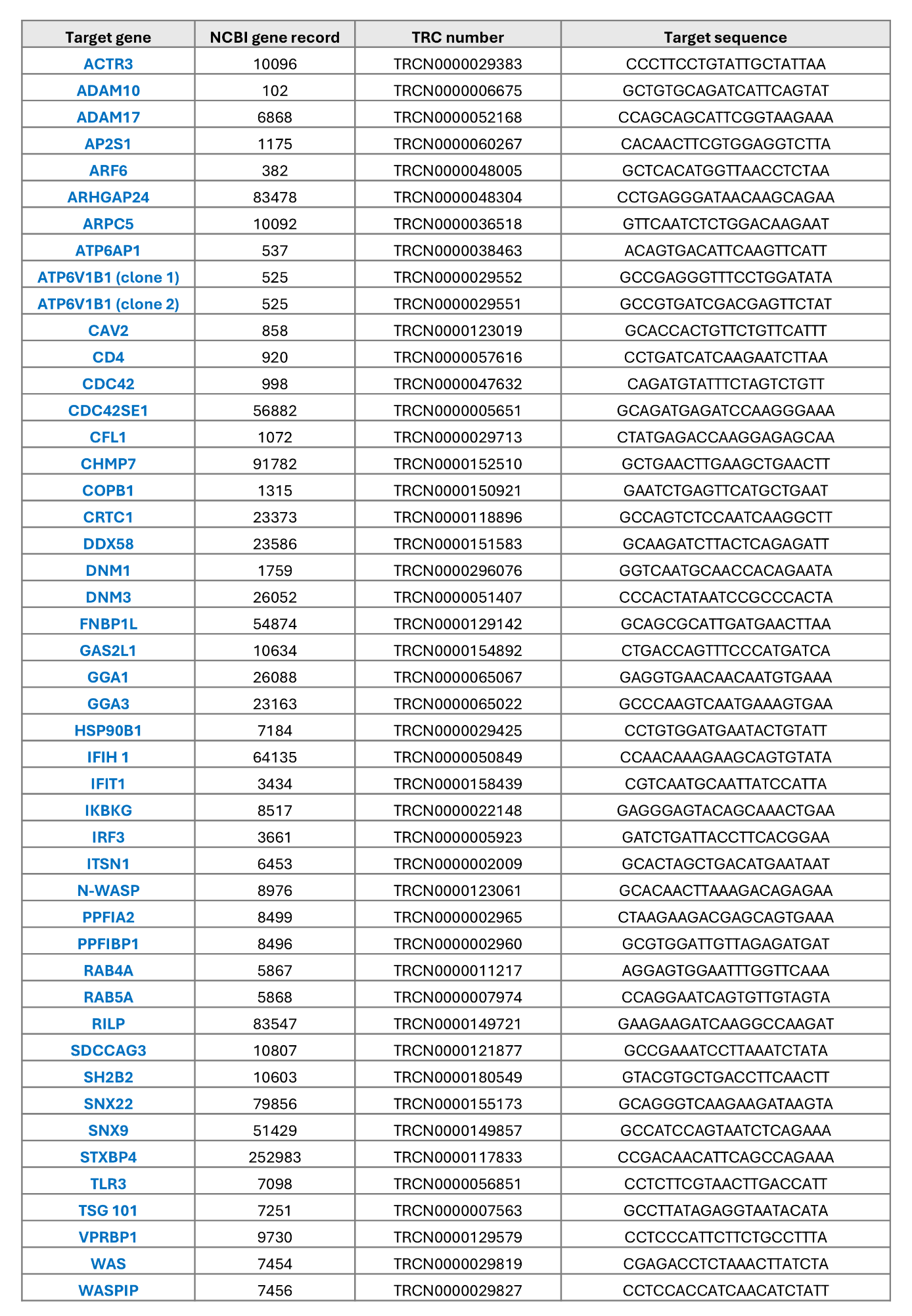
**

**Supplemental Table 1 List of shRNA sequences used during the study.**
